# More is less: Effect of ICF-based early progressive mobilization on severe aneurysmal subarachnoid hemorrhage in the NICU

**DOI:** 10.3389/fneur.2022.951071

**Published:** 2022-12-14

**Authors:** Xiaolong Yang, Lei Cao, Tiantian Zhang, Xin Qu, Wenjin Chen, Weitao Cheng, Meng Qi, Na Wang, Weiqun Song, Ning Wang

**Affiliations:** ^1^Department of Rehabilitation Medicine, Xuanwu Hospital, Capital Medical University, Beijing, China; ^2^Intensive Care Unit, Department of Neurosurgery, Xuanwu Hospital, Capital Medical University, Beijing, China

**Keywords:** early mobilization, severe aneurysmal subarachnoid hemorrhage, intensive care unit, international classification of functioning, disability and health, external ventricular drain, neurocritical illness

## Abstract

**Introduction:**

Aneurysmal subarachnoid hemorrhage (aSAH) is a type of stroke that occurs due to a ruptured intracranial aneurysm. Although advanced therapies have been applied to treat aSAH, patients still suffer from functional impairment leading to prolonged stays in the NICU. The effect of early progressive mobilization as an intervention implemented in the ICU setting for critically ill patients remains unclear.

**Methods:**

This retrospective study evaluated ICF-based early progressive mobilization's validity, safety, and feasibility in severe aSAH patients. Sixty-eight patients with aSAH with Hunt-Hess grades III-IV were included. They were divided into two groups—progressive mobilization and passive movement. Patients in the progressive mobilization group received progressive ICF-based mobilization intervention, and those in the passive movement group received passive joint movement training. The incidence of pneumonia, duration of mechanical ventilation, length of NICU stay, and incidence of deep vein thrombosis were evaluated for validity. In contrast, the incidence of cerebral vasospasm, abnormally high ICP, and other safety events were assessed for safety. We also described the feasibility of the early mobilization initiation time and the rate of participation at each level for patients in the progressive mobilization group.

**Results:**

The results showed that the incidence of pneumonia, duration of mechanical ventilation, and length of NICU stay were significantly lower among patients in the progressive mobilization group than in the passive movement group (*P* = 0.031, *P* = 0.004, *P* = 0.012), but the incidence of deep vein thrombosis did not significantly differ between the two groups. Regarding safety, patients in the progressive mobilization group had a lower incidence of cerebral vasospasm than those in the passive movement group. Considering the effect of an external ventricular drain on cerebral vasospasm (*P* = 0.015), we further analyzed those patients in the progressive mobilization group who had a lower incidence of cerebral vasospasm in patients who did not have an external ventricular drain (*P* = 0.011). Although we found 2 events of abnormally increased intracranial pressure in the progressive mobilization group, there was no abnormal decrease in cerebral perfusion pressure in the 2 events. In addition, among other safety events, there was no difference in the occurrence of adverse events between the two groups (*P* = 0.073), but the number of potential adverse events was higher in the progressive mobilization group (*P* = 0.001). Regarding feasibility, patients in the progressive mobilization group were commonly initiated 72 h after admission to the NICU, and 47.06% were in the third level of the mobilization protocol.

**Discussion:**

We conclude that the ICF-based early progressive mobilization protocol is an effective and feasible intervention tool. For validity, more mobilization interventions might lead to less pneumonia, duration of mechanical ventilation and length of stay for patients with severe aSAH in the NICU, Moreover, it is necessary to pay attention over potential adverse events (especially line problems), although we did not find serious safety events.

## 1. Introduction

Aneurysmal subarachnoid hemorrhage (aSAH) is a neurologic disease associated with high mortality and morbidity, leading to primary brain lesions, hydrocephalus, delayed cerebral ischemia (DCI), and other serious disease complications. Especially, patients with severe aSAH (Hunt-Hess grade ≥III) require mechanical ventilation (MV) and admission into the Neuro Intensive Care Unit (NICU) (1, 2). In the last decades, with the use of advanced technology in the ICU, a modest improvement has been noticed in the survival rate of patients. However, it is a seesaw-like situation for patients referred to critical care. We must ensure that the patient's outcome is tilted toward the better side to battle the problems that may occur during the ICU stay. Several studies have pointed out that early mobilization and rehabilitation of critically ill patients in the ICU can improve their functional capacity and exert a positive effect on hospital outcomes (3). Whether these benefits apply to neurologically critically ill patients remains unclear, as neuro ICU patients are often excluded from clinical trials due to patients' specific neurologic symptoms and overall medical condition. Undeniably, neurocritical patients are affected by multiple factors, such as prolonged MV, impaired neurologic function, and immobility. Patients often suffer from lower physical activity, poor respiratory function, pneumonia, difficult weaning, ICU-acquired weakness (ICU-AW), and many other complications seriously affecting the outcome (4). Current evidence suggests early mobilization and rehabilitation is a promising intervention to improve patients' functional recovery at hospital discharge (5, 6); however, few studies support the use of this intervention in severe aSAH patients.

The International Classification of Functioning, Disability, and Health (ICF), a core member of the World Health Organization Family International Classifications, covers four components: body structures and functions, activity and participation, environmental, and personal factors. A theoretical framework based on the “bio-psycho-social” functioning model can systematically and comprehensively guide the application of rehabilitation services (7, 8). This study evaluates the validity, safety, and feasibility of ICF-based early progressive mobilization in patients with severe aSAH in the NICU. It provides suggestions for early rehabilitation interventions in neurocritical patients.

## 2. Materials and methods

### 2.1. Participants

This retrospective study included 68 patients with aSAH admitted to the neurosurgical ICU of XW Hospital of Capital Medical University from December 2019 to October 2021. Diagnosis of aSAH was established based on sudden clinical symptoms and imaging diagnostic criteria (9). All patients were given nimodipine, a calcium channel blocker drug. The patients were divided into two groups—progressive mobilization and passive movement, with 34 patients in each group. The inclusion criteria were as follows: (1) first presentation and diagnosis of aSAH; (2) single aneurysm and repair; (3) Hunt-Hess grade III-IV; (4) being mechanically ventilated and NICU treatment >48 h duration; (5) cough reflex present. The exclusion criteria were as follows: (1) potential risk of aneurysm rupture; (2) previous history of central nervous system disease; (3) having been bedridden for ≥3 months before this admission; (4) unstable fracture or deep vein thrombosis; (5) malignancy or pregnancy.

### 2.2. Interventions

Both groups received conventional treatment, including maintenance and monitoring of vital clinical signs, nursing care, and nutritional support. The passive movement group received passive joint movement training, while the progressive mobilization group implemented ICF-based early progressive mobilization based on conventional clinical treatment. All the mobilization procedures were performed by physiotherapists with >3 years of experience in critical care rehabilitation, once a day for five consecutive days a week until the patients were discharged from the NICU.

Passive movement training treats the passive joints to restore limb movements. Passive movement is performed at the patient's shoulder, elbow, wrist, hand, hip, knee, ankle, and foot within their joint range of motion, 12–15 times/group, 2–3 groups/parts, once a day, and 5 days/week.

**Figure 1:**
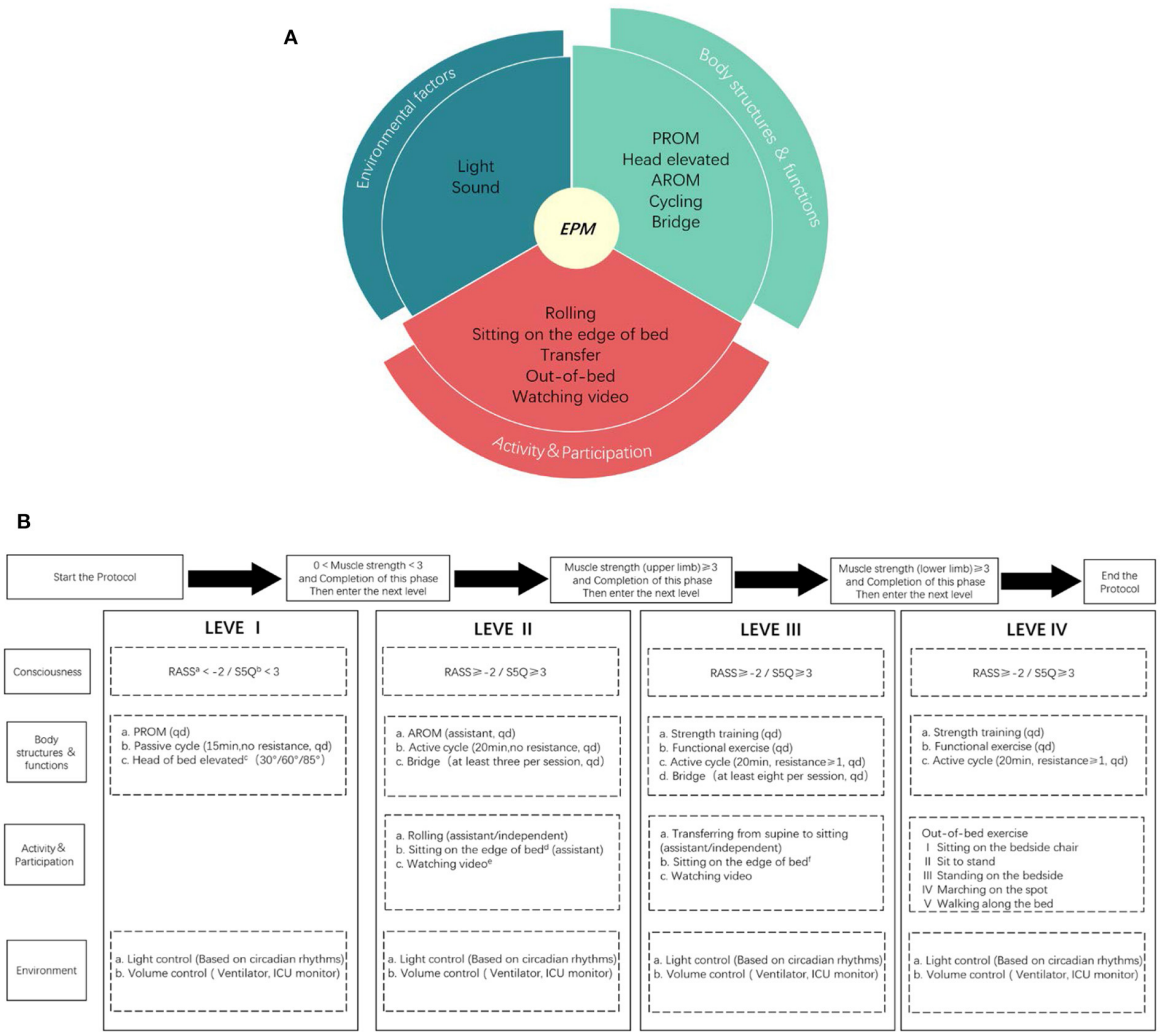
ICF based progressive mobilization interventions **(A)** and the protocol **(B)**. EPM means early progressive mobilization; AROM means active range of motion; PROM means passive range of motion; Muscle strength is allowed on either limb, without requiring both limbs; The environmental interventions are adjusted without affecting the clinical treatment; a: RASS means Richmond Agitation Sedation Scale; b: S5Q means Standardized Five Questions; c: Head elevation in three steps, each step is done for at least 1 h/day, each step takes at least 1 day and the patient is observed to tolerate the change in position before moving on to the next step; d: Sitting on the edge of bed at least 20 min a day, requiring the patient's upper body to leave the bed and at least one person to assist, with the patient supporting a walker or bed block to maintain balance and placing a support under feet; e: Watching video means videos recorded by the family or something the patient enjoyed; f: Sitting on the edge of the bed at least 20 min a day, the upper body of the patient should leave the bed and at least one person should be protected without touching, the patient can support a walker or bed block to maintain balance but no support placed under feet.

Progressive mobilization protocol is another training session developed by a multidisciplinary early mobilization team (including ICU physicians, rehabilitation physicians, physical therapists, and nurses). This protocol was based on the protocols of Morris et al. (10) and Karic et al. (5) and implemented a multisystemic intervention for better physical function, activity and participation, and environmental factors under the guidance of the ICF framework. The early mobilization protocol was progressively adjusted by evaluating the patient's cooperation and muscle strength, including head elevation, motor function training, cycling, out-of-bed training, etc. The training intensity was gradual and patient-tolerated, once a day, 5 days/week (Figure 1).

Stop criteria are applied to patients in both groups when they experience cardiovascular, respiratory, or neurological instability and abnormal monitoring values that were not caused by device malfunction during mobilization. The intervention was immediately stopped, and patients in the progressive mobilization group were reevaluated and sent back to the previous level of the mobilization protocol. It follows (1) Heart rate (HR) that differs more than 20% from the resting level; (2) Blood pressure (BP) ≥20 mmHg; (3) Respiratory rhythm (RR) >40 breaths/min, or < 5 breaths/min; (4) SpO_2_ < 88% and longer than 3 min; (5) symptomatic cerebral vasospasm or new neurologic events; (6) not following the directions in case of abnormal mental status; (7) intolerance due to discomfort, diaphoresis, or trembling; (8) malignant arrhythmia.

### 2.3. Outcomes

Patient's clinical characteristics data including age, gender, intracranial pressure (ICP) monitoring number, the duration of external ventricular drainage (EVD), Hunt-Hess grade (III/IV), aneurysm location (anterior circulation, posterior circulation), and aneurysm repair (neurosurgical clipping or endovascular coiling) were observed. The validity evaluations included incidences of occurrence of pneumonia, duration of mechanical ventilation, length of NICU stay, and incidence of deep vein thrombosis (DVT) and the safety evaluations included incidence of cerebral vasospasm (CVS), the incidence of abnormal increase of ICP, and cerebral perfusion pressure (CPP). In addition, the study provided the information on the stage of early mobilization of patients present in the progressive mobilization group they were discharged from the NICU and the initiation time of early mobilization for patients in each level to explore further the feasibility of early mobilization in the NICU for patients with severe aSAH. The evaluation methods and contents are as follows.

#### 2.3.1. Validity

##### 2.3.1.1. Incidence of pneumonia

Two ICU physicians diagnosed pneumonia by examining the patient's clinical symptoms, imaging, and laboratory findings such as fever, chest radiography, leukocytosis, purulent secretions, positive cultures of endotracheal aspirates, or hypoxemia.

##### 2.3.1.2. Duration of mechanical ventilation

The duration of MV was measured in days, and the time spent between the use of MV and weaning in the NICU was recorded (if the patient was not weaned at the time of transfer from the NICU, the time of transfer was used). Successful weaning was defined as a patient who met the signs of weaning and passed the spontaneous breathing test (SBT), a negative leak test, and a smooth spontaneous breathing process after weaning without excessive involvement of the auxiliary respiratory muscles maintained for at least 30 min. The signs of weaning were stable breathing, well-oxygenation (FiO_2_ ≤ 0.5, PEEP ≤ 10 cmH_2_O, Pa0_2_/FiO_2_ ≥ 200 mmHg), hemodynamically stable, and rapid shallow breathing index (RSBI) < 105 breaths/min/L.

##### 2.3.1.3. Length of stay

LOS was measured in days, and the time spent by patients from the time of admission to discharge from NICU was recorded.

##### 2.3.1.4. Incidence of deep vein thrombosis

DVT was diagnosed by ultrasound on the day of admission to the NICU and then weekly until discharge, the DVT occurrence in all patients from ICU admission to discharge was recorded.

#### 2.3.2. Safety

##### 2.3.2.1. Incidence of cerebral vasospasm

Cerebral vasospasm was identified using transcranial Doppler (TCD) ultrasound. Considering that EVD may affect the occurrence of CVS in patients with aSAH, in this study, the incidence of CVS in patients with and without EVD in each group was recorded according to the use of EVD in both groups. The effect of mobilization and EVD on the occurrence of CVS was described further by Multivariate logistic regression analysis.

##### 2.3.2.2. Incidence of abnormally increased ICP

ICP monitoring was performed using an external ventricular drain along with a connected hydraulic sensor (ICP-EVD), and ICP was recorded during the mobilization intervention (the drainage tube had to be clamped during the mobilization, and the drainage tube was opened, and the tube condition was promptly observed after mobilization). An abnormally increased ICPs was defined as an ICP value > 20 mmHg (11) and because ICP monitoring was not done in all patients in both groups. This study separately recorded the total number of mobilization interventions (for each progressive mobilization or passive movement intervention, a record was made) and the number of abnormally increased ICP during the monitoring period. Meanwhile, considering that abnormally increased ICP may lead to decreased cerebral perfusion, the study also recorded the changes that occurred in CPP in patients with abnormally increased ICP (CPP = MAP–ICP). CPP < 60 mmHg was defined as abnormally decreased CPP (12).

##### 2.3.2.3. Other safety events

The total number of mobilizations (or each progressive mobilization or passive movement intervention) was recorded. The number of other safety events and the incidence (number of other safety events/total number of mobilization) was recorded separately for both groups. Other safety events were defined as events other than CVS and abnormally increased ICP that resulted or potentially resulted in any adverse changes in the patient's clinical status because of the mobilization (progressive mobilization/passive movement) intervention, including events that met the stop criteria. Safety events occurred during and within 1 h after each mobilization intervention occurred in patients from both groups. They were also classified into adverse events and potential adverse events according to the severity of their influence on the patients. Adverse events are events that cause hemodynamic abnormalities or direct harm, including invasive lines dislodgement (endotracheal or tracheostomy tube, transfusion catheter, urinary catheter, and feeding tube), falls, cardiovascular-related events (HR changed over 20%, BP changed over 20 mmHg, arrhythmia, cardiac arrest, orthostatic hypotensive), respiratory-related events (tachypnea, desaturation < 88%), and other neurologic-related events (changes in consciousness, seizures). Potential adverse events are referred to as events that did not directly harm the patients. Still, they would indirectly affect the patient's monitoring and management, including tracheostomy tube out of midline, non-invasive lines dislodgement (cardiac monitoring lines, thermometry tube, oximetry finger cuff), feeding tube unfastens, as well as not following directions for various reasons.

#### 2.3.3. Feasibility

##### 2.3.3.1. Participation rate of each level

We recorded the stage of each patient in the progressive mobilization protocol when the patients in the progressive mobilization group were discharged from the NICU, expressed as the participation rate (number of patients in each level/total number of patients in the progressive mobilization group).

##### 2.3.3.2. Early mobilization initiation time

The initiation time is the time difference between the patient's first engagement in progressive mobilization protocol and the patient's admission to the NICU, recorded in hours. And the mobilization initiation time of patients at each level was further analyzed according to the level of the progressive mobilization protocol where each patient was discharged from the NICU in the progressive mobilization group.

### 2.4. Statistics

Data were analyzed using SPSS 23.0 software (IBM, USA) quantitative data were tested for normality using the Shapiro-Wilk methods. Values were expressed as mean ± standard deviation ($\bar{x}$ ± s) for those conforming to a normal distribution, non-normally distributed quantitative data were described using median and quartiles [M (P25, P75)], and qualitative data were expressed as cases (%). Two independent samples *t*-test was used to compare groups of quantitative data conforming to a normal distribution. Mann-Whitney *U*-test was used to compare the groups of quantitative data with non-normal distribution. The Chi-square test or Fisher's exact test was used to compare groups of qualitative data. The differences were statistically significant at *P* < 0.05.

## 3. Results

### 3.1. Clinical characteristics of the subjects

Sixty-eight aSAH patients were included in this study. We found no statistically significant differences in age, gender, number of ICP monitoring cases, duration of EVD, Hunt-Hess grade (III/IV), aneurysm location (anterior circulation, posterior circulation), and aneurysm management (surgical cranial clamping, endovascular interventional embolization) among them (Table 1).

**Table 1:** Patients characteristics of the two groups.

	**Progressive mobilization group (*n* = 34)**	**Passive movement group (*n* = 34)**	***P*-value**
Age (years)	68.32 ± 6.06	69.65 ± 5.91	0.365
ICP monitoring, *n* (%)	13 (38.24)	15 (44.12)	0.622
Duration of EVD (days)	4 (3.50, 5.50)	4 (3, 6)	0.964
**Genders**, ***n*** **(%)**
Male	11 (32.35)	14 (41.18)	0.451
Female	23 (64.71)	20 (58.82)	
**Hunt-Hess grade**, ***n*** **(%)**
III	22 (64.71)	21 (61.76)	0.801
IV	12 (35.29)	13 (38.24)	
**Aneurysm location**, ***n*** **(%)**
Anterior circulation	19 (55.88)	18 (52.94)	0.808
Posterior circulation	15 (44.12)	16 (47.06)	
**Aneurysms repair**, ***n*** **(%)**
Neurosurgical clipping	9 (26.47)	8 (23.53)	0.779
Endovascular coiling	25 (73.53)	26 (76.47)	

ICP, intracranial pressure; EVD, external ventricular drainage.

### 3.2. Comparison of validity

**Figure 2:**
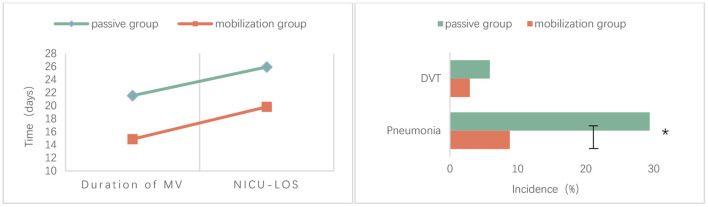
Comparison of validity between the two groups; *mean *p* < 0.05.

The proportion of pneumonia was significantly lower in the progressive mobilization group (8.82%) than in the passive movement group (29.41%) (*P* = 0.031). The mean duration of mechanical ventilation was significantly lower in the progressive mobilization group (14.88 ± 7.12 days) than in the passive movement group (21.53 ± 10.88 days) (*P* = 0.004). The mean LOS in NICU was significantly lower in the progressive mobilization group (19.82 ± 8.13 days) than in the passive movement group (25.91 ± 11.00 days) (*P* = 0.012). However, there was no significant difference in the incidence of DVT between the progressive mobilization group (2.94%) and the passive movement group (5.88%) (*P* > 0.05; Table 2, Figure 2).

**Table 2:** Comparison of validity between the two groups.

	**Progressive mobilization group (*n* = 34)**	**Passive movement group (*n* = 34)**	**Diff. 95% confidence interval**	***P*-value**
Pneumonia, *n* (%)	3 (8.82)	10 (29.41)	20.59% [2.55–38.63%]	0.031
Duration of MV (days)	14.88 ± 7.12	21.53 ± 10.88	6.65 [2.18–11.11]	0.004
NICU-LOS (days)	19.82 ± 8.13	25.91 ± 11.00	6.09 [1.40–10.78]	0.012
DVT, *n* (%)	1 (2.94)	2 (5.88)	NA	1.000

MV, mechanical ventilation; NICU-LOS, Length of stay in Neuro Intensive Care Unit; DVT, deep vein thrombosis; NA, Not Applicable.

### 3.3. Comparison of safety

#### 3.3.1. Incidence of cerebral vasospasm

With CVS as a dependent variable and progressive mobilization (progressive mobilization, no progressive mobilization) and EVD (EVD, no EVD) as independent variables, the results showed that progressive mobilization and EVD were the influencing factors for patients with severe aSAH (Table 3).

**Table 3:** Multivariate logistic regression analysis of cerebral vasospasm.

	***N***	**Odds ratio (95% confidence interval)**	***P*-value**
**Progressive mobilization**			
No	34	1	
Yes	34	0.200 (0.058–0.691)	0.011
**EVD**
No	40	1	
Yes	28	0.219 (0.059–0.819)	0.024

EVD, external ventricular drainage.

Nineteen patients suffered from CVS, and all showed non-symptomatic CVS, including five cases in the progressive mobilization group and 14 cases in the passive movement group. The incidence of CVS was significantly lower in the progressive mobilization group (14.71%) than in the passive movement group (41.18%) (*P* = 0.015). Among the patients who implemented EVD, there was no significant difference in the incidence of CVS between the progressive movement group (7.69%) and the passive movement group (20.00%) (*P* > 0.05). For the patients who did not implement EVD, the incidence of CVS was significantly lower in the progressive mobilization group (19.05%) than in the passive movement group (57.89%) (*P* = 0.011; Table 4).

**Table 4:** Comparison of neuro-safety events between the two groups.

	**Progressive mobilization group**	**Passive movement group**	**Diff. 95% confidence interval**	***P*-value**
Total NOP	34	34	NA	NA
Total CVS incidence, *n* (%)	5 (14.71)	14 (41.18)	26.47% [6.09–46.85%]	0.015
NOP in EVD group	13	15	NA	NA
CVS incidence, *n* (%)	1 (7.69)	3 (20.00)	NA	0.600
NOP in non-EVD group	21	19	NA	NA
CVS incidence, *n* (%)	4 (19.05)	11 (57.89)	38.85% [11.01–66.68%]	0.011
Number of mobilizations within ICP	58	67	NA	NA
Abnormally Increased ICP, *n* (%)	2 (3.45)	0	NA	0.213
Abnormally decreased CPP, *n* (%)	0	0	NA	NA

NOP, number of patients; EVD, external ventricular drainage; ICP, intracranial pressure; CPP, cerebral perfusion pressure; NA, Not Applicable.

#### 3.3.2. Incidence of abnormally increased ICP

In the progressive mobilization group, patients who received ICP monitoring (13 cases) had 58 mobilization interventions during the monitoring period, there was two events of abnormal increase in ICP, but no CPP < 60 mmHg was found in either event. For the passive movement group, patients who received ICP monitoring (15 cases) had 67 mobilization interventions during the monitoring period, there was no abnormal increased ICP occurred. We found no significant difference in the incidence of abnormally increased ICP between the progressive movement group (3.45%) and the passive movement group (0%) (*P* = 0.213; Table 4).

#### 3.3.3. Other safety events

During the intervention, the total number of mobilizations in the progressive mobilization group was 417. There were 26 safety events (6.24%), except for CVS and abnormally increased ICP, including 8 adverse events (four invasive lines dislodgement, two cardiovascular-related events, two respiratory-related events), 18 potential adverse events (six tracheostomy tube out of midline, nine non-invasive lines dislodgement, one feeding tube unfasten and two not following directions). For the passive movement group, the total number of mobilizations was 511. There were eight other safety events (1.57%), including three adverse events (two invasive lines dislodgement, one cardiovascular-related event), and five potential adverse events (two tracheostomy tubes out of midline, three non-invasive lines dislodgement).

The total incidence of other safety events was significantly higher in the progressive mobilization group (6.24%) than in the passive movement group (1.57%) (*P* < 0.01). in particular, the incidence of potential adverse events was significantly higher in the progressive mobilization group (4.32%) than in the passive movement group (0.98%) (*P* < 0.01), Among the potential adverse events, only the events of “non-invasive lines dislodgement” was significantly higher in the progressive mobilization group (2.16%) than in the passive movement group (0.59%) (*P* = 0.042). However, for the incidence of adverse events, there was no significant difference between the progressive movement group (1.92%) and the passive movement group (0.59%) (*P* = 0.073; Table 5).

**Table 5:** Comparison of other safety events between the two groups.

	**Progressive mobilization group**	**Passive movement group**	***P*-value**
Number of mobilizations	417	511	NA
Number of other safety events, *n* (%)	26 (6.24)	8 (1.57)	0.0002
**Adverse events**, ***n*** **(%)**	8 (1.92)	3 (0.59)	0.073
**Invasive lines dislodgement**	4 (0.96)	2 (0.39)	0.417
Ventilator tube	1 (0.24)	1 (0.20)	1
Transfusion catheter	2 (0.48)	1 (0.20)	0.591
Urinary catheter	1 (0.24)	0	0.449
**Falls**	0	0	NA
**Cardiovascular events**
HR changed	2 (0.48)	1 (0.20)	0.591
BP changed	0	0	NA
Arrhythmia	0	0	NA
Cardiac arrest	0	0	NA
Orthostatic hypotensive	0	0	NA
**Respiratory events**
Tachypnea	2 (0.48)	0	0.202
Desaturation	0	0	NA
**Other neurologic events**
Changes in consciousness	0	0	NA
Seizures	0	0	NA
**Potential adverse events**, ***n*** **(%)**	18 (4.32)	5 (0.98)	0.001
**Tracheostomy tube out of midline**	6 (1.44)	2 (0.39)	0.150
**Non-invasive lines dislodgement**	9 (2.16)	3 (0.59)	0.042
Cardiac monitoring lines	3 (0.72)	1 (0.20)	0.332
Thermometry tube	1 (0.24)	0	0.449
Oximetry finger cuff	5 (1.20)	2 (0.39)	0.253
**Feeding tube unfasten**	1 (0.24)	0	0.449
**Not following directions**	2 (0.48)	0	0.202

P-value is the value of Fisher's exact test; NA, Not Applicable.

### 3.4. Comparison of feasibility

**Figure 3:**
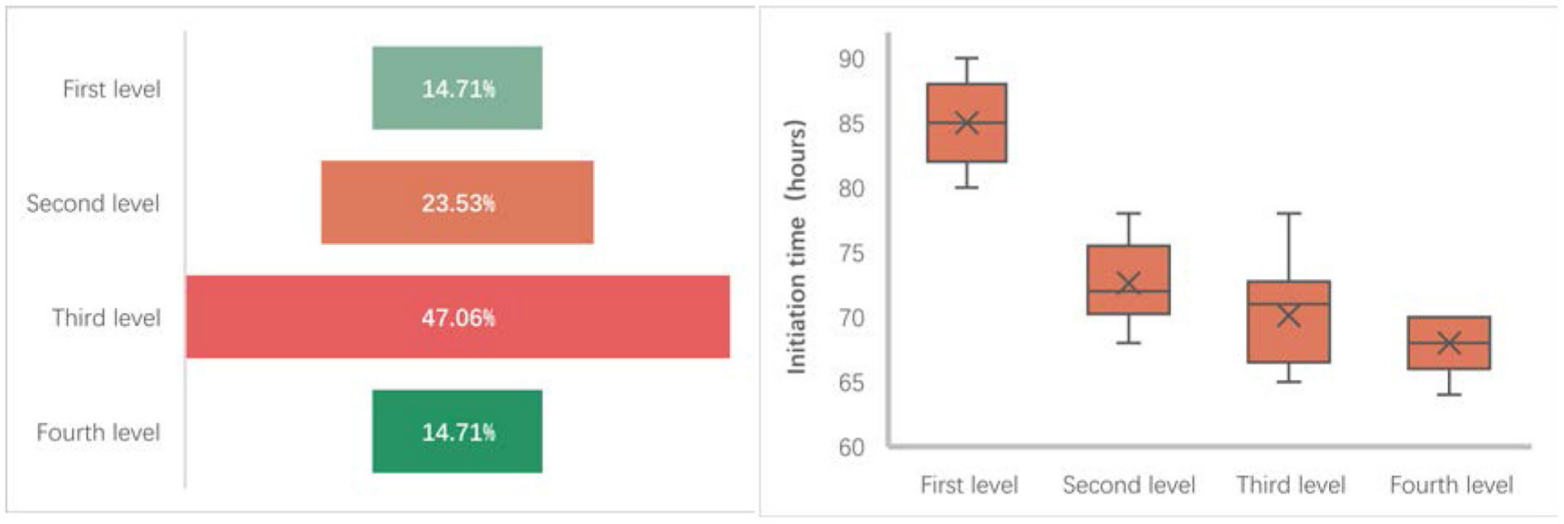
Participation rate and initiation time in mobilization group.

During the progressive mobilization protocol of 34 patients in the progressive mobilization group, nearly half of the patients (16 cases) were in the third level of the mobilization protocol when they were discharged from the NICU, five were in the first level, eight were in the second level, and five were in the fourth level. From the timing of the progressive mobilization group, the initiation time of the early progressive mobilization intervention in the progressive mobilization group was generally concentrated around 72 h after the patients had been admitted to the NICU, the shortest average initiation time for patients who were in the fourth level when they were discharged from the NICU (68 h after admission to the NICU), the longest average initiation time for patients who were in the first level (85 h after admission to the NICU), and the average initiation time for patients in the second and third level was 72 and 70 h, respectively (Figure 3).

## 4. Discussion

Our early progressive mobilization protocol was based on the ICF framework guidelines, which contain joint movement, cycling, and out-of-bed exercises, mostly implemented on critically ill patients. Under the ICF framework's guidance, the mobilization protocol's implementation was systematically applied to all the patients suffering from severe aSAH in NICU based on three aspects (body structures and functions, activity and participation, environmental factors). We found that more mobilization interventions might lead to fewer complications and benefit the patient, such as less incidence of pneumonia, less duration of mechanical ventilation, and LOS for severe aSAH patients in NICU. In addition, we also found less severe safety issues in early progressive mobilization.

### 4.1. Pneumonia, duration of mechanical ventilation, and LOS in NICU

Systemic complications are common in patients with SAH; however, during hospitalization, the incidence of acquired pneumonia is about 20% (13), and for severe SAH, it could be even higher. In critically ill patients, pneumonia persists throughout MV. It is considered ventilator-associated pneumonia (VAP) occurring *via* endotracheal intubation while receiving MV > 48 h, LOS in the NICU, or leading to a worse clinical prognosis (14, 15). Our study found that patients in the progressive mobilization group had a lower incidence of pneumonia (8.82%) with a shorter duration of mechanical ventilation and length of NICU stay than the passive movement group.

Although the conventional care given to both the groups has provided passive turning (1 time/2 h) and mechanically assisted sputum evacuation (2 times/d). More patients in the progressive mobilization group (70.59%) participated in active mobilization, such as bedside sitting and out-of-bed exercises, which reduced the adverse effects of bed rest on pulmonary function (16, 17). We speculated that this anti-gravity position had some beneficial effects; however, further validation is needed. In addition, it has been suggested that the psychological status of critically ill patients might affect the treatment outcomes (18, 19) and also considered that the surrounding physical environmental stimulation devices such as lights and alarms present in the ICU environment may further lead to psychological problems such as anxiety and fear (20, 21). As a part of psychological support, positive encouragement during mobilization interventions and improvement of one's abilities by maintaining self-confidence to achieve a better recovery might play a key role in a patient's recovery (22, 23). The ICF-based progressive mobilization protocol has not only included environmental adjustments like day/night alternating light adjustment and alarm tone size setting but also provided videos recorded by family members or out of patients' usual interest, which may influence the patient's psychological state. However, we could not evaluate the patient's psychological state due to the lack of data. In particular, the “bias” of the implementers, who were the core members of the multidisciplinary early mobilization groups during the implementation of the progressive mobilization protocol, has shown an impact on the attitudes during implementation (24, 25), even on the validity of the early mobilization interventions. Although this aspect was not investigated in this study, our experience suggests that the effective participation of implementers would be a “special factor” in facilitating the mobilization implementation.

### 4.2. Deep vein thrombosis

Deep vein thrombosis is the most common complication observed in patients with aSAH, especially in critically ill patients who are often immobilized in bed. The intake of antifibrinolytic drugs is also an important factor causing deep vein thrombosis. Although antifibrinolytic drugs have remained controversial, existing studies have recommended that clinicians recognize deep vein thrombosis early and prevent it in advance.

This study found that DVT incidence was lower in both groups. The difference remained insignificant, which may be due to high vigilance and prevention of DVT in our center. However, all the patients were required to be screened for DVT on the first admission to the NICU. We found that none of the patients had DVT on initial screening. Later, a preventive combination of balloon compression and compression stockings was applied. However, there was no clear evidence to confirm the effect of combined preventive therapy on the incidence of DVT in patients with severe aSAH, but considering the adverse impact of DVT, most guidelines and research have recommended applying this approach to patients with aSAH (26, 27). These confounding factors that cannot be excluded could be the low incidence of DVT in both groups. Our findings were consistent with those of Klein et al. (28); for neurocritical illness patients, due to the complex causes of DVT in critically ill aSAH patients, more subgroup analysis studies are needed in the future.

### 4.3. Cerebral vasospasm

Safety concerns have been a barrier to implementing early mobilization in critically ill patients. Numerous studies have demonstrated that early mobilization is safe for patients who are not neuro-critically ill (29). But for patients who are neuro-critically ill patients, brain-protection-based management, and neurologic monitoring have made the implementation of early mobilization more careful (30). For patients with aSAH, cerebral vasospasm is often observed in the initial days after aneurysm rupture (31). Cerebral vasospasm may also lead to DCI development, the most life-threatening complication (32). Therefore, to avoid the adverse effects of early mobilization on cerebral vasospasm, further implementation of early mobilization was restricted in severe aSAH patients. However, bed rest does not effectively prevent the occurrence of cerebral vasospasm (33). Based on previous studies, postural changes that occurred in patients with SAH did not confer any significant effect on the cerebral blood flow velocity and regional cerebral blood flow (34, 35), and the safety of implementing early mobilization in neuro-critically ill patients using EVD was reported efficiently (36–38). Both have supported the implementation of early mobilization, but most studies did not emphasize the safety of implementing an early mobilization in patients with severe aSAH (Hunt-Hess grade ≥ III).

The implementation of early mobilization remained significant in reducing the incidence of CVS (14.71 vs. 41.18%, with a difference of 26.47%). Through Multivariate logistic regression analysis, we found that EVD and progressive mobilization intervention were protective factors on CVS in patients with severe aSAH, which further showed the role of progressive mobilization in reducing the incidence of CVS. However, patients in both groups in this study also used EVD, considering the effect of EVD on the incidence of CVS. Therefore, we further divided the patients into EVD and non-EVD groups by comparing the incidence of CVS in the two groups without EVD. The results also showed that the incidence of CVS was lower in the progressive mobilization (19.05 vs. 57.89%, with a difference of 38.85%), which may be benefited by the positive drainage effect of postural changes on cerebrospinal fluid during progressive mobilization (5, 39, 40). Interestingly, although there was no significant difference in the number of patients and days of drainage between the two groups using EVD in this study and no significant difference between the groups, the incidence of CVS was lower in the progressive mobilization group than in the passive movement group (7.69 vs. 20.00%, with a difference of 12.31%), which might be due to the smaller sample size in the EVD group. In addition, it is important to mention that early mobilization in patients with aSAH using EVD, despite being safe, still needs to be highly concerned about the impact of mobilization implementation on EVD, which not only depends on the experience and carefulness of the mobilization implementers but also the cultural environment of the multidisciplinary team for early mobilization (e.g., training, communication, and collaboration) (41) may be an equally critical factor for safety.

### 4.4. Increased ICP

Increased ICP is a “characteristic” safety event in neurocritical patients, leading to serious complications such as hydrocephalus if overlooked (1). Head elevation of 30° can lead to a decrease in ICP in patients with increased ICP after aSAH and is now used as a general measure to decrease the cranial pressure (42, 43), but few studies have confirmed the effect of other postural mobilization on ICP. In this present study, by monitoring ICP in patients with severe aSAH during mobilization (progressive mobilization, passive movement), the results showed that during the 58 mobilization times in the progressive mobilization group had received ICP monitoring, two patients had one event of abnormally increased ICP (ICP values of 23–25 mmHg in EVD clamping) in each of the second levels of the protocol during turning. However, when we stopped turning and elevated the head at 30°, the ICP decreased to normal after 5 min without affecting the drug dose. No cerebral ischemic events (DCI) occurred in these two patients during their NICU stay.

In addition, two patients in our study showed abnormally high ICP without any abnormal decrease in CPP (< 60 mmHg), accompanied by a short-term increase in MAP, which could be attributed to the patient's brain modulation. Although we could not explore further, this would probably tell us from the side that the brain of patients with severe aSAH may not be as bad as we thought, so we did not report the MAP change as an adverse event. Harrois et al. (44) showed that a patient's abnormally increased ICP might be associated with higher baseline ICP values. In our study, we observed that two patients had higher baseline ICP values than the others (18–20 mmHg in the EVD clamping). Thus, in terms of safety, although our study found a low incidence of abnormally increased ICP in patients with severe aSAH due to early mobilization intervention, we cannot yet deny the adverse effect of turning training on ICP in patients with severe aSAH, considering the risk of abnormally increased ICP. Therefore, it is recommended that patients with severe aSAH implementing early mobilization (especially when turning and other postural mobilization) should pay attention to the baseline number of ICP and consider the impact it may cause to develop appropriate management strategies.

### 4.5. Other safety events

Currently, the definition of safety events for early mobilization is inconsistent, despite many studies demonstrating a low incidence of safety events (3, 14, 45, 46) and have mostly focused on abnormalities in hemodynamic indicators, which would produce serious safety concerns; however, most abnormal changes in hemodynamic indicators may not be associated with early mobilization. Considering the characteristics of early mobilization and neurocritical patients, we described the safety events, excluding the abnormal increase of CVS and ICP separately. Also, we classified other safety events into adverse and potential adverse events according to their effects on patients. The total number of mobilizations (417 vs. 511), adverse events (1.92 vs. 0.59%), and potential adverse events (4.32 vs. 0.98%) were also recorded for patients from the progressive mobilization group and passive movement group. Results showed that compared with the passive movement group, patients in the progressive mobilization group were more likely to have potential adverse events due to diverse mobilization interventions. However, they did not lead to direct safety issues. However, considering the indirect effects and the “high” incidence of potential adverse events, more attention should be paid to mobilization.

The incidence of adverse events in this study was low (1.92%), but invasive lines dislodgement occurred during mobilization in both groups (0.96 vs. 0.39%), which occurred mainly due to the force of pulling on the lines by limb movement. Although the incidence is low, the mobilization implementers need to check and place the lines before the intervention is initiated, whether the active mobilization intervention such as out-of-bed is implemented. For potential adverse events, it was shown that patients in the progressive mobilization group had a high incidence (4.32%), accounting for up to 69.23% of other safety events in this group of patients. The difference was significant compared with patients in the passive movement group. This is mainly due to the risk that occurs during turning or out-of-bed exercise during progressive mobilization is more likely to cause lines problems such as tracheostomy tube out of midline or dislodgement than passive movement in bed. Moreover, even though patients in the passive movement group did not implement the position interventions, the intervention appeared simpler and safer. There were still five potential adverse events, especially two tracheostomy tubes out of midline, mainly due to pulling on the tracheostomy tube while performing passive joint movements, which suggested that the tracheostomy tube in MV patients may be a problem that limited the movement of the upper limb joints. The mobilization implementers need to manage the lines before the passive joint movement.

Additionally, if critical care patients have feeding tubes fastened with tape, they are often not well-fastened due to sweating, and the oximetry finger cuff is often dropped or poorly fastened during the implementation of mobilization, causing abnormal alarms. Thus, it is important to pay attention to patient monitoring and the fastening of various types of lines before and during the implementation of mobilization. Appropriate “line handling” will reduce the safety events of early mobilization of neurocritical patients.

There was also one patient in this study who was not following directions during two mobilizations due to agitation, which led to the discontinuation of the mobilization. This event was recorded as a potential adverse event because it resolved after rest and did not affect the patient's sedation medication, which further showed that for neurocritical patients, mental status, delirium, and cognitive impairment may affect the patient's mobilization implementation and should be focused more closely.

In general, although we suggested that early intervention is safe in patients with severe aSAH, safety concerns should still be considered key factors in the implementation of mobilization. Adequate preparation and monitoring before the implementation of mobilization programs are important. It should be noted that the treatment of critically ill patients relies on systematic observation and comprehensive inference, not just the pursuit of “standard numbers.” Therefore, we suggested that to improve the safety of early mobilization implementation, the safety standards should be set under the surveillance and participation of the ICU physicians. The observation and emergency response abilities of the implementers should be highly regarded.

### 4.6. Definition and feasibility

Currently, there is no consistency in the definition of early mobilization. Some studies have defined it as a mobilization intervention that starts within 7 days of admission to the ICU (47) while others as a mobilization intervention that begins within 72 h of ICU admission (3). Schaller et al. (48) suggested that the early start of mobilization is the key to a successful outcome; hence, we agree that the timing of early mobilization initiation is important, but it is still a subject of debate as to what is the ideal time for initiation. For that, many studies are needed to confirm the effect of different initiation timings on early mobilization interventions, especially in neurocritical patients. Otherwise, to make the definition consistent, we suggested including the ICU setting in the definition of early mobilization. No matter how early it is, the mobilization interventions implemented in the ICU setting for the stage of critical illness are defined as early mobilization. In this study, the initiation time of early mobilization was generally around 72 h after admission to the NICU for patients in the progressive mobilization group. The average initiation time was shorter for patients in level IV (68 h) of the mobilization protocol and longer for patients in level I (85 h), relating to the disease dynamics of patients with severe aSAH. However, the initiation time of early mobilization depends on the clinician's decision regarding the patient's condition and the decision of the multidisciplinary team of early mobilization, which is influenced by the “culture” of the ICU, staffing, and device, which can be a barrier or a facilitator in the implementation of mobilization (49).

Regarding the feasibility, Hodgson et al. (50) suggested key strategies for optimizing the early mobilization and rehabilitation in the critical care phase, which provided an important direction for early mobilization. To further describe the feasibility of mobilization implementation, we recorded the level of mobilization at which patients in the progressive mobilization group were discharged from the NICU. The results showed that most patients (70.59%) progressed to levels II and III by the time they were discharged from the NICU. For patients with severe aSAH, the out-of-bed exercises (transferring, standing, and walking) in level IV were more demanding on patients' motor functions. However, 14.71% of patients progressed to level IV, which mainly depended on the severity of lesions in functional areas of the brain. Unfortunately, we could not complete the prognostic follow-up as we were unsuccessful in contacting most of the patients. Nevertheless, all patients showed better adaptation during the mobilization intervention. Thus, compared to non-neurologic critically ill patients, aSAH patients are limited due to their brain impairment, which hampers mobilization implementation. Still, our study suggested that a structured, progressive mobilization protocol based on the ICF can be systematically applied to critically ill patients in the ICU environment while adjusting for the environmental factors. Early mobilization of patients with aSAH with this systematic, structured, progressive protocol can reduce the barriers to early mobilization, and improve the feasibility of implementation.

## 5. Conclusion

Severe aSAH is an acute cerebrovascular disease that seriously damages the central nervous system. This study showed that the implementation of progressive mobilization protocol was feasible in the NICU; in terms of validity, the progressive mobilization was effective in reducing the incidence of pneumonia and shortening the duration of mechanical ventilation and LOS in the NICU, but it did not affect deep vein thrombosis. The results showed that the ICF-based progressive mobilization protocol has demonstrated good safety and can reduce the incidence of CVS in patients with severe aSAH. For aSAH patients on EVD, even though mobilization may lead to an abnormal increase in ICP, it is rare, transient, and manageable and may also be related to the high baseline ICP. However, there is still a higher incidence of potential adverse events with progressive mobilization than in the passive movement group, including tracheostomy tube out of midline and non-invasive lines dislodgement. We found that more mobilization interventions might lead to lower issues. We expect more studies and advanced technologies to help these groups of patients have better outcomes in the future.

## Data availability statement

The raw data supporting the conclusions of this article will be made available by the authors, without undue reservation.

## Ethics statement

The studies involving human participants were reviewed and approved by Ethics Committee of Xuanwu Hospital [The Teaching Hospital Affiliated with Capital Medical University; approval number (2019)032]. The patients/participants provided their written informed consent to participate in this study.

## Author contributions

XY and LC designed the experiments. XY analyzed the data and drafted the manuscript. WS and NiW supervised the research. All authors formed a multidisciplinary early mobilization team and contributed to the article and approved the submitted version.
